# Circumferential dissection of deep fascia as ancillary technique in circumcision: is it possible to correct phimosis increasing penis size?

**DOI:** 10.1186/s12894-021-00782-y

**Published:** 2021-02-03

**Authors:** Pagano Carmine, Faenza Mario, Guastafierro Antonio, Manfellotto Vincenzo, Grella Elisa, Cosenza Angelo, Pieretti Gorizio, Izzo Sara

**Affiliations:** 1 Clinica Nostra Signora di Lourdes, Somma, Italy; 2 Multidisciplinary Department of Medical Surgical and Dental Sciences - Plastic Surgery Unit, Università degli Studi della Campania “Luigi Vanvitelli”, Caserta, Italy; 3 Department of Advanced Medical and Surgical Sciences - General Surgery Unit, Università degli Studi della Campania “Luigi Vanvitelli”, Caserta, Italy

## Abstract

**Background:**

Phimosis is the inability to retract the preputium downward over the glans penis. Despite the various techniques of preputial plasty described in literature, the most performed surgical treatment is still the conventional circumcision.

**Methods:**

In this paper we retrospectively reviewed data of a homogeneous population of 36 consecutive adult patients who underwent phimosis correction by circumcsion with dissection of the Deep Fascia. Patients were followed up by one independent plastic surgeon that measured penis length and circumference in nonerected state preoperatively and at 6 month time postoperatively.

**Results:**

The Wilcoxon Signed Rank Test showed a significant (*p* < 0.0001) difference between the two groups both in terms of length and circumference.

**Conclusions:**

In conclusion, the ancillary technique we described leads to an increase of penis size, is safe and easy to perform and does not increase significantly operative time nor complication rate to the conventional procedure.

## Background

Phimosis is a clinical condition in which the prepuce cannot be retracted over the glans. In pediatric population it is common and can be considered physiologic, whereas in adult population is always considered pathologic and is related to many conditions such as diabetes, or recurrent balanitis or trauma [1, 2]. Phimosis had been classified by Kayaba et al. [3] in four grades according to the retractability of the prepuce.

The most performed surgical treatment of this clinical condition is circumcision but alternative surgical techniques with preputial preservation have been described [4–6].

The chronic inflammatory conditions that lead to phimosis in adults are often associated with a certain grade of fibrosis, from simple glanular adhesions to constricting bands affecting a larger portion of penile skin.

These fibrotic reactions could consequently lead to a reduction in penis size due to chronic and progressive constriction [12].

During the last 30 years augmentative surgery of penis has gradually increased its appeal to the point of being considered equivalent to the need of some women to ask for breast augmentation [18].

In this paper we described an ancillary procedure in circumcision that can lead to an augmentation both in terms of penile length and circumference.

## Methods

We retrospectively reviewed data of a homogeneous population of 36 consecutive adult patients, aged between 19 and 45 years old, who underwent phimosis correction between November 2018 and March 2019. All patients were classified in terms of phimosis grade, symptoms and characteristics (Table 1). No patients reported history of previous surgery or scarring at prepuce level.

**Table 1:** The deep fascia

Characteristics	Patients n = 36
Age (years old)	19–45 (average 23,7)
Kayaba’s grade 1	6 (16.66%)
Kayaba’s grade 2	19 (52.77%)
Kayaba’s grade 3	11 (30.55%)
Smoking habit	11 (30.55%)
Diabetes	8 (22.22%)
History of recurrent balanoposthitis	6 (16.66%)
Painful erections	15 (41.66%)
Postoperative complications	
Huge edema	3 (8.33%)
Sensory impairments	10 (27.77%)

Written informed consent was obtained from all participants.

A two-step surgical procedure was performed under local anaesthesia obtained by peripheral block of the dorsal penile nerve with 2% Xylocaine.

**Fig. 1:**
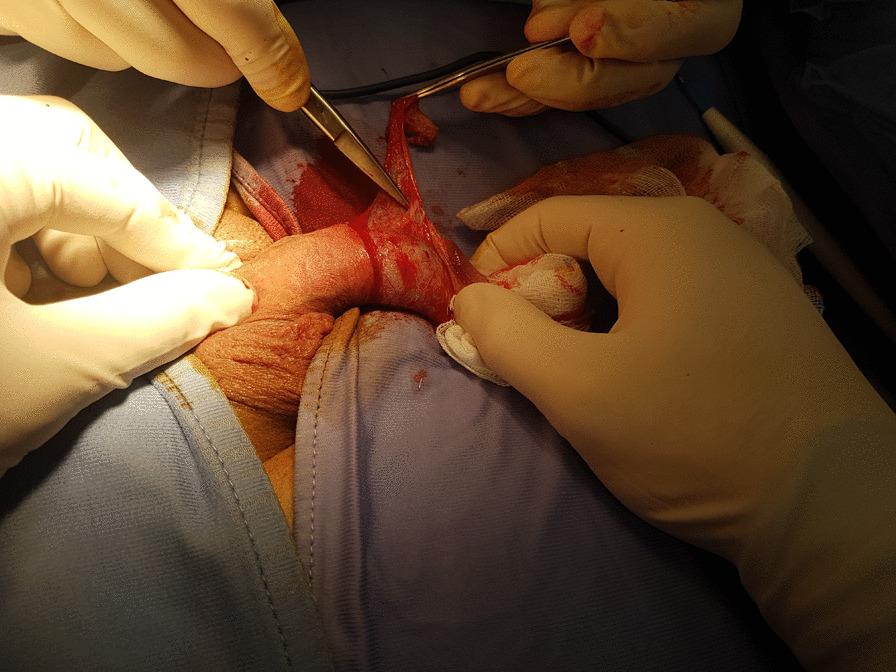
Removal of the constricting fibrous band and consensual penile frenulectomy

The first step consisted in a conventional circumcision (Fig. 1).

**Fig. 2:**
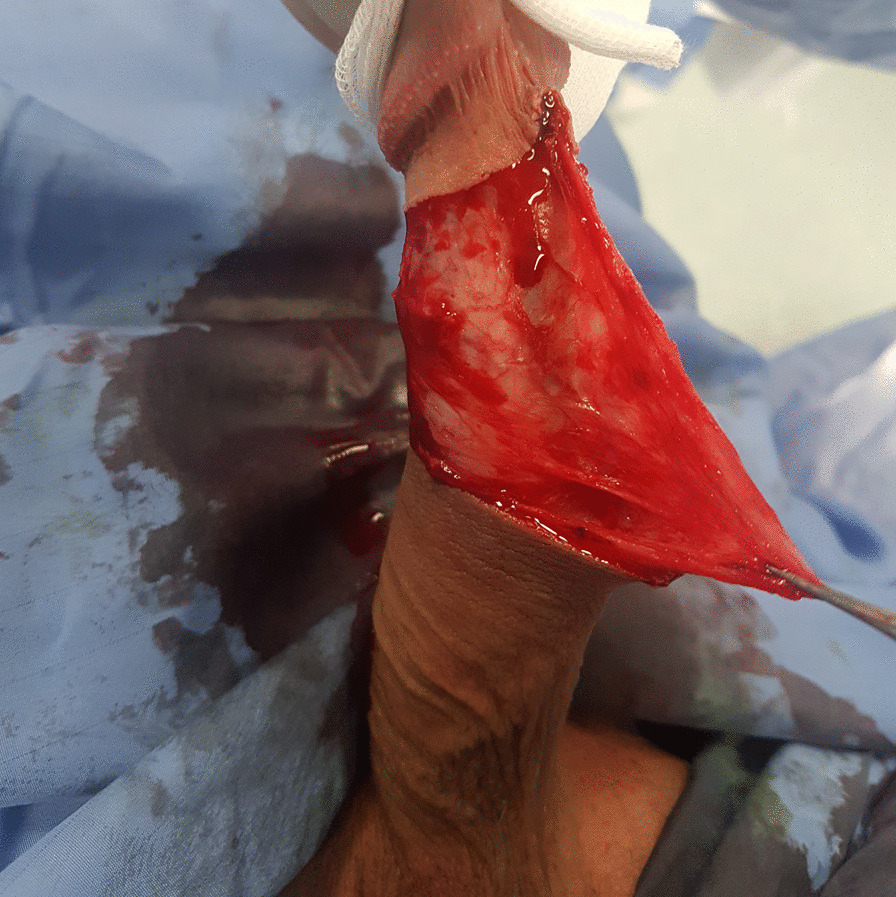
Skin of the penis body was retracted to the base, showing ventrally the presence of the areolar tissue and deep fascia

In the second step the skin of the penis body was retracted to the base showing ventrally the presence of the areolar tissue and Buck’s Fascia (Fig. 2).

**Fig. 3:**
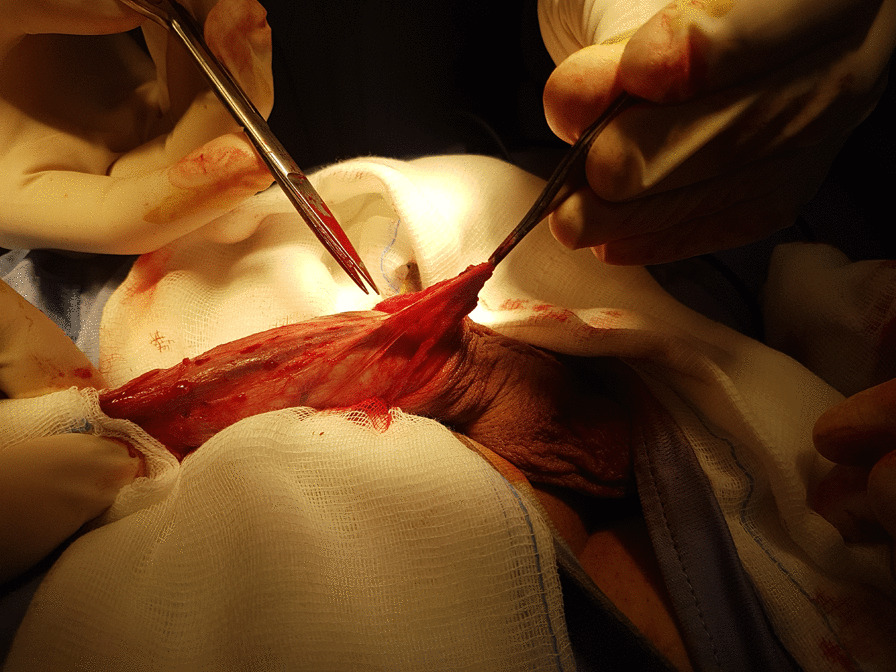
Adherent bridles were identified and carefully dissected

**Fig. 4:**
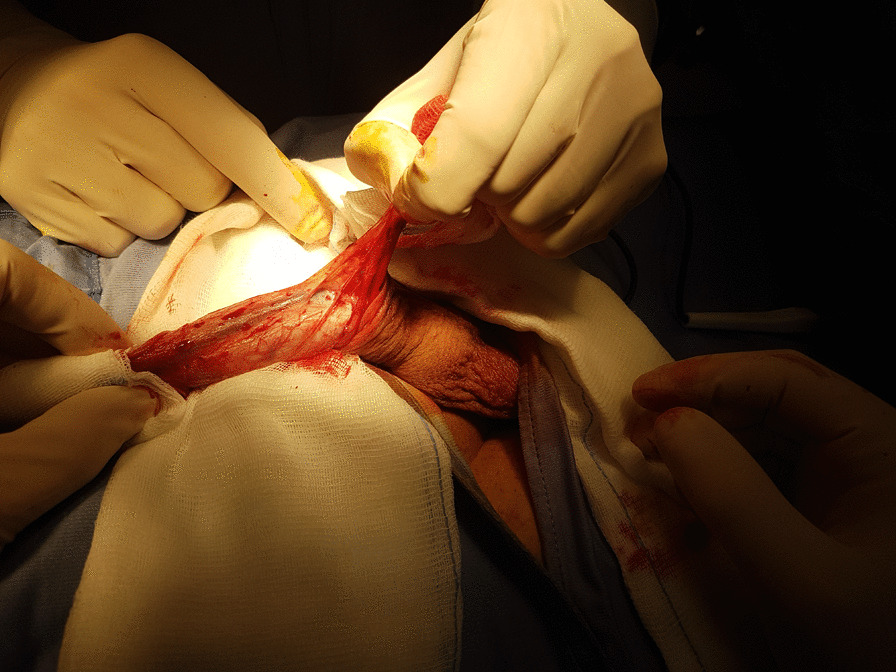
With the ventral aspect of the penis exposed, a manual traction was performed, causing a partial relaxation of the deep fascia, determining the expansion of the corpora cavernosa and the spongeous body of the urethra

Adherent bridles were identified and bluntly dissected (Fig. 3). With the ventral face of the penis exposed, a manual traction was performed causing a partial relaxation of the Buck’s Fascia and determining the expansion both of the corpora cavernosa and the spongious body of the urethra (Fig. 4). At the end of the surgery, the entire cut edges were approximated with interrupted fine absorbable stitches.

## Results

Patients were followed up by one independent plastic surgeon that measured the stretched penis length from radix to the apex of glans and circumference at midpoint of the penile shaft in nonerected state after 10 min of acclimatization at room temperature preoperatively and at 6 month time postoperatively.

In 8 patients out of 36 (22.22%) we registered a gain only in terms of length, whereas in 5 patients out of 36 we found an increment only in terms of circumference.

Only 1 patient out of 36 did not show any dimensional change (Table 2).

**Table 2:** Deep fascia the expansion

Patients	Circumference (cm)			Lenght (cm)		
	Pre-op	6 months Post-op	Gain	Pre-op	6 months Post-op	Gain
1	9.7	10.5	0.8	9.8	11	1.2
2	9	10.5	1.5	9.2	9.5	0.3
3	10.1	11.5	1.4	7.2	9.5	2.3
4	12	12	0	9.5	10.5	1
5	8.5	11.5	3	10.5	12.5	2
6	8.5	10	1.5	10.5	10.5	0
7	9.2	10.5	1.3	9.5	9.8	0.3
8	11.5	11.5	0	12.5	13	0.5
9	11	11	0	11.5	12	0.5
10	9	10.5	1.5	10	11	1
11	8.5	10	1.5	8.5	9.5	1
12	9.5	10.5	1	8.5	9	0.5
13	8	9.5	1.5	7	8	1
14	8.5	9.5	1	10	10.5	0.5
15	10	10.3	0.3	10	10	0
16	10.5	11.5	1	11	11.5	0.5
17	9	10.5	1.5	9.2	9.5	0.3
18	12	12	0	9.5	10.5	1
19	8.5	10	1.5	10.5	10.5	0
20	11	11	0	11.5	12	0.5
21	8.5	10	1.5	8.5	9.5	1
22	8.5	9.5	1	10	10.5	0.5
23	10	10.5	0.5	10	10	0
24	9	10.5	1.5	9.2	10	0.8
25	9	10.5	1.5	9	9.5	0.5
26	9.5	11	1.5	8	9.3	1.3
27	11	11	0	9.5	10.5	1
28	9.5	11.5	2	10.5	11	0.5
29	9	10	1	10.5	10.5	0
30	8.4	9.5	1.1	9.5	10	0.5
31	11.5	11.5	0	12	13	1
32	10	10	0	11	12	1
33	9	9.5	0.5	10	11	1
34	10	10	0	8.5	8.5	0
35	9.5	10.8	1.3	8.5	9	0.5
36	8.5	9	0.5	9	11	2

The Wilcoxon Signed Rank Test showed a significant (*p* < 0.0001) difference between the two groups both in terms of length and circumference. The mean of gain of circumference was 0.95 + 0.71 cm, while the mean for gain in length was 0.7 + 0.56 cm.

The only complications registered were due to huge edema in 3 patients and sensory impairment in 10 patients occurred postoperatively and spontaneously regressed in 2 weeks time.

No recurrence of phimosis at 6 months follow-up have been registered.

## Discussion

Phimosis is a clinical condition in which the foreskin, due to adherences or fibrotic preputial ring, cannot be pulled back over in order to expose the glans penis.

Phimosis can be classified into two groups: congenital and acquired.

The first type is usually seen in young children and could be considered physiological.

Acquired phimosis is mostly seen in adolescents and adults. Apart from discomfort during coitus in adult life, if the distal preputial ring is too narrow it can cause ballooning during voiding, making it difficult to maintain penile hygiene leading to chronic balanoposthitis.

This chronic condition leads to the development of adhesions between the glans and the inner leaf of the preputium and also the development of a fibrotic ring, severely narrowing the opening of the prepuce.

In 1996 Kayaba et al. [3] classified phimosis condition in four grades according to the level of preputial retractability.

Type I the preputial retractability is totally absent.

In Type II the preputial opening allows only exposure of the external urethral meatus.

In Type III the preputium can be partially retracted from the apex to the middle of the glans.

In Type IV the preputium can be retracted allowing the exposure to above the crown of the glans, because of adhesions between the inner leaf of the preputium and the corona.

Despite the various techniques of preputial plasty described in literature [4–6], the most performed surgical treatment is still the conventional circumcision.

This should be preferred if the preputium becomes scarred from previous attempts to release the glans or in the presence of balanitis xerotica obliterans.

Differing from reconstructive surgery, cosmetic surgery was developed by surgeons especially for people who felt the need to improve on nature, to improve the results of previous injuries or interventions by surgery.

Until 30 years ago, penis size either nonerected or erected was not mentioned in literature.

Cosmetic surgery for the penis cannot and should not be compared to rhinoplasty, breast augmentation, or breast reduction surgery, where obtained results are generally very acceptable.

Unfortunately, the expectations of patients of augmentation phalloplasty are far greater than the real results that can be obtained with the current surgical techniques.

When performed on a normal-looking penis which has an average size, a penile cosmetic procedure yields aesthetically less than the desired result. An increase of 2–4 cm should be considered a success.

The historical Kinsey report showed that only 5% of men have an erection of less than 9 cm and that 1% are very well endowed with an erection of longer than 20 cm [7].

A more recent study by Ponchietti et al. [8] of penile size in 3300 young Italian men showed these differences in a large sample: flaccid penile length: 5–13 cm (average 9.0 cm), stretched penile size: 7.5–17.5 cm (average 12.5 cm), flaccid penile circumference: 8.5–11.5 cm (average 10 cm).

Another study by Wessels et al. [9] found that the average length of a flaccid penis was 8.8 cm, stretched length 12.4 cm and erect length 12.9 cm.

Khan et al. [10] asserted that men referred with penile disease had a marginally shorter penile length. In his study, men affected by penile disease, showed a flaccid length of 8.37 cm, a penopubic length of 9.97 cm and a stretched length of 13.70 cm.

In literature several surgical techniques have been described for penis augmentation, such as section of the suspensory ligament of the penis, lipectomy or liposuction of prepubic fat, inverted V-plasty of the radix of the penis and fat grafting of the penis [11].

Anyway is widely understood how a penis enlargement surgical or non surgical procedure is highly contraindicated in patients affected by uncorrected penile pathological conditions.

In 2011 Montag and Palmer [12] published a review about abnormalities of penile curvature and size, stating that these conditions can be related both to phimosis itself, because of the thickening of the Buck’s fascia, and also to scar contraction after circumcision.

Many factors such as genetic predisposition, history of non-gonococcal urethritis, smoking habit, fibrotic lesions of the genital tract or previous urologic surgical procedures can lead to formation of asymptomatic fibrous cords at the level of penile fascia, with various degrees of contraction [13–15].

The suspensory apparatus of the penis is part of the deep fascia of the abdominal wall, it consists in the fundiform ligament, the suspensory ligament proper and the arcuate subpubic ligament.

The fundiform ligament is superficial and not adherent to the tunica albuginea, whilst the suspensory ligament proper bridges between the symphysis pubis and the Dartos and Buck’s fascia of the corpora cavernosa, it splits to surround the penis and then unites and blends inferiorly with the Dartos’ fascia forming the scrotal septum.

In our paper we described an ancillary technique in circumcision procedure in which we performed a release of the areolar tissue of the preputial skin, inducing a relaxation of the adherences of the Buck’s fascia of penis.

In this way the corpora cavernosa are decompressed and, in our opinion, that is the reason why the circumference of the penis is significantly lengthened after the procedure.

The technique we described does not differ in its rationale from scar revision and nerve decompression procedures [16, 17].

For what concerns the gain in terms of penile length, the rationale should be related to the fact that Buck band is in continuity with the suspensory ligament of the penis, consequently its surgical release determines an elongation of the penis shaft.

After surgery the mean of gain of circumference observed was 0.95 + 0.71 cm, while in terms of length was 0.7 + 0.56 cm.

These data are comparable to the results obtained by other penis enlargement techniques, but to date there is no evidence of a contraction of the buck band in healthy patients, and therefore there is no evidence that this operation can lead to an increase in penis size even in healthy patients.

Further studies will be necessary to verify these premises in order to determine the real effectiveness of the technique also in penile cosmetic surgery.

## Conclusions

In conclusion, the ancillary technique we described is safe and easy to perform and does not increase significantly operative time, since the degloving procedure takes just 15 min, nor complication rate to the conventional procedure.

For these reasons it could be done in every adult patient affected by phimosis, regardless of grade, who undergoes circumcision procedure.

## Data Availability

All data generated or analysed during this study are included in this published article [and its supplementary information files].
